# Evaluation of Feline Renal Perfusion with Contrast-Enhanced Ultrasonography and Scintigraphy

**DOI:** 10.1371/journal.pone.0164488

**Published:** 2016-10-13

**Authors:** Emmelie Stock, Katrien Vanderperren, Tim Bosmans, André Dobbeleir, Luc Duchateau, Myriam Hesta, Lien Lybaert, Kathelijne Peremans, Eva Vandermeulen, Jimmy Saunders

**Affiliations:** 1 Department of Medical Imaging of Domestic Animals, Faculty of Veterinary Medicine, Ghent University, Salisburylaan 133, Merelbeke, Belgium; 2 Department of Medicine and Clinical Biology of Small Animals, Faculty of Veterinary Medicine, Ghent University, Salisburylaan 133, Merelbeke, Belgium; 3 Ghent University Hospital, Department of Radiology and Nuclear Medicine, De Pintelaan 185, 9000 Ghent, Belgium; 4 Department of Comparative Physiology and Biometry, Faculty of Veterinary Medicine, Ghent University, Salisburylaan 133, Merelbeke, Belgium; 5 Department of Nutrition, Genetics and Ethology, Faculty of Veterinary Medicine, Ghent University, Salisburylaan 133, Merelbeke, Belgium; 6 Lab of Pharmaceutical technology, Faculty of Pharmaceutical Sciences, Ghent University, Ottergemsesteenweg 460, Ghent, Belgium; Colorado State University, UNITED STATES

## Abstract

Contrast-enhanced ultrasound (CEUS) is an emerging technique to evaluate tissue perfusion. Promising results have been obtained in the evaluation of renal perfusion in health and disease, both in human and veterinary medicine. Renal scintigraphy using ^99m^Tc-Mercaptoacetyltriglycine (MAG_3_) is another non-invasive technique that can be used to evaluate renal perfusion. However, no data are available on the ability of CEUS or ^99m^Tc- MAG_3_ scintigraphy to detect small changes in renal perfusion in cats. Therefore, both techniques were applied in a normal feline population to evaluate detection possibilities of perfusion changes by angiotensin II (AT II). Contrast-enhanced ultrasound using a bolus injection of commercially available contrast agent and renal scintigraphy using ^99m^Tc-MAG_3_ were performed in 11 healthy cats after infusion of 0,9% NaCl (control) and AT II. Angiotensin II induced changes were noticed on several CEUS parameters. Mean peak enhancement, wash-in perfusion index and wash-out rate for the entire kidney decreased significantly after AT II infusion. Moreover, a tendency towards a lower wash-in area-under-the curve was present. Renal scintigraphy could not detect perfusion changes induced by AT II. This study shows that CEUS is able to detect changes in feline renal perfusion induced by AT II infusion.

## Introduction

Chronic kidney disease is of major importance in both human and veterinary medicine with a prevalence of 13% in people and even up to 50% in randomly selected cats, increasing to 68,8% in cats with degenerative joint disease [1–3]. It is a progressive disorder with often an unclear etiology. Early diagnosis is essential as intervention in early stage of the disease process provides better life expectancy and quality [4]. The assessment of renal perfusion is an important component in the evaluation of kidney disease as these renal perfusion changes occur in early state of the disease progress [5].

However, performing an accurate, noninvasive measurement of renal perfusion is challenging. The gold standard to estimate renal plasma flow is determination of para-amino hippuric acid (PAH) clearance, although this is technically laborious and requires specific equipment, limiting the use in clinical circumstances.

Renal perfusion can also be evaluated using radioactive tracers: ortho-iodo-hippuric acid (OIH) and mercaptoacethyltriglycerine (MAG_3_). Both tracers have high first-pass extraction rate, but do not reach an extraction rate of 100%, thus the term ‘effective renal plasma flow’ (ERPF) is used. Ortho-iodo-hippuric acid has a similar chemical structure to PAH, and can be bound to ^123^I or ^131^I. Although the disadvantage is poor quality images compromising quantification, the tracer is still useful for blood clearance techniques [6]. Mercaptoacetyltriglycine can be labeled with ^99m^Tc, making it a good tracer for imaging procedures [6]. Despite its 30% lower clearance compared to OIH, high agreement between both tracers was found in both humans and dogs [7, 8]. Significant hepatic uptake of MAG_3_ is noted in cats, thus requiring camera-based investigation as they allow distinction of the separate organs. Some limitations are associated with the use of radioactive tracers, including costs, involvement of radiation, and limited equipment availability.

Contrast-enhanced ultrasound is a functional ultrasound technique that allows assessment of both macro- and microcirculation. Ultrasound contrast agent consists of tiny gas-filled bubbles (microbubbles) that have a rheology similar to red blood cells after intravenous injection. It is an extremely safe, cost-effective technique, which does not involve the use of ionizing radiation [9, 10]. The rate of adverse effects is close to zero, and, in contrast to iodinated contrast agents used in computed tomography or gadolinium-based contrast agent used in magnetic resonance imaging, no nephrotoxicity is involved, allowing safe use in geriatric and pediatric patients and patients with renal insufficiency [9]. Several studies in human medicine have shown promising results for the use of CEUS in the diagnosis of diffuse renal disorders, such as early assessment of chronic kidney dysfunction, diabetic kidney damage, and rejection of renal transplants [11–15]. In dogs, CEUS was proven to be useful for the early detection of iatrogenic chronic ischemic renal disease and to detect diffuse renal changes in beagles with iatrogenic hypercortisolism [16, 17]. Nevertheless, it remains unclear if small changes in renal perfusion can be detected using a bolus injection of ultrasound contrast agent in cats.

The objective of this study was to investigate the ability of CEUS and renal scintigraphy using ^99m^Tc-MAG_3_ to detect changes in renal perfusion of healthy cats at baseline and during infusion of angiotensin II (AT II, an arterial vasoconstrictor).

## Materials and Methods

### Animals

The study was carried out in strict accordance with the recommendations of the European Convention for the Protection of Vertebrate Animals used for Experimental and other Scientific Purposes. The protocol was approved by the Ethical Committee of the Faculty of Veterinary Medicine of Ghent University (EC2014/38). All efforts were made to minimize suffering. During the experiments, the cats were continuously monitored by experienced veterinarians to assess basic clinical parameters and signs of discomfort. Since suffering was limited and no detrimental effect on further life quality was present, the cats are further kept as experimental animals.

Eleven healthy purpose-bred European Shorthair cats (Charles River Laboratories (30/3202), France and Lab of animal nutrition (LA2400378), Ghent university, Belgium) without any history of cardiovascular, renal or endocrine disease were included. They were judged healthy based on physical examination and non-invasive Doppler blood pressure measurement, hematology, biochemistry profile, urinalysis, and abdominal ultrasound. The cats were between 4 and 8 years (5.29 ± 1.25 years), with a body weight ranging from 2.4 and 5.1 kg (3.53 ± 0.78 kg; body condition score 4-5/9). Eight cats were female, 3 were male, and all of them were neutered. The cats were housed in indoor in stable groups of 10 cats, with free access to water, and fed a standard dry food twice a day.

### Study design

The cats were premedicated with butorphanol (Dolorex, 10 mg/ml, MSD animal health) 0.2 mg/kg IV, 20 minutes before anesthesia induction. Anesthesia was induced with propofol (Propovet^®^, 10 mg/ml, Abbott Laboratories) IV given to effect (6.30 ± 1.13 mg/kg), until endotracheal intubation could be performed. Anesthesia was maintained with isoflurane vaporized in 100% oxygen, using a non-rebreathing system, to reach an end tidal isoflurane percentage of 1.2–1.4%.

All the subjects received a vasoconstrictor (AT II) and a control treatment (0.9% sodium chloride), in a randomized order, with a washout period of 14 days. Vasoconstriction was obtained with an infusion of AT II at a rate of 2 ng/kg/min using a syringe pump (Perfusor Space, BBraun, Germany) for the total duration of the CEUS study and renal scintigraphy. Cats undergoing control treatment received an infusion of sterile 0.9% sodium chloride at the same rate and total volume as the AT II infusion. Infusion was started 5 minutes after the start of the inhalation anesthesia. On each study day, renal perfusion was evaluated using CEUS and MAG_3_ scintigraphy, started 15 minutes after initiation of the intravenous infusion of AT II or 0.9% sodium chloride. The imaging techniques were performed in a randomized order.

Blood pressure (non-invasive Doppler measurement), heart rate, peripheral arterial oxygen saturation, end tidal isoflurane and carbon dioxide concentrations were closely monitored.

### Preparation angiotensin II

A stock solution (1000 μg/mL) was prepared by adding 1 mg of anhydrous angiotensin II (Stigma-Aldrich, USA) to 1 mL of sterile 0.9% sodium chloride solution. This solution was filtered through a 0.22 μm filter (Steriflip Vaccuum Filtration System with millipore Express PLUS membrane, Millipore Corporation, Belgium), aliquoted into sterile cryovials (100 μL per vial), and stored at –80°C. On the study day, 1 aliquot was thawed on ice and serially diluted in sterile 0.9% NaCl solution to produce solutions with final concentration of 0.1 μg/mL.

### Contrast-enhanced ultrasound

The hair was clipped over the ventrolateral portion of the abdomen. Alcohol and coupling gel were applied to the skin. The US exams were performed with the cat in dorsal recumbency.

The left kidney was centered on the screen and imaged in a longitudinal plane. The transducer was manually positioned by the same person during each imaging procedure and was maintained at the same position during the CEUS examination.

A 0.15 mL bolus of sulfur hexafluoride-filled microbubbles (Sonovue^®^, Bracco, Italy) was injected into a 22-gauge indwelling catheter in the cephalic vein. The same person performed the bolus injection in a standardized way in all cats. The ultrasound contrast agent was injected over approximately 3 seconds followed by injection of 1 mL saline bolus. A three-way stopcock was used to minimize any delay between the injection of microbubbles and saline. Three injections of contrast were performed: the first injection was not used for further evaluation. Between subsequent injections, remnant microbubbles were destroyed in the caudal abdominal aorta by setting the acoustic power at the highest level during 2 minutes.

All examinations were performed using a linear transducer of 12–5 MHz on a dedicated machine (iU22, Philips, Bothell, WA) with contrast-specific software. Basic technical parameters were a high dynamic range setting (50 dB), single focus placed directly under the kidney, persistency off, mechanical index 0.09, timer started at the beginning of the injection. For the gain setting, we started with a nearly dark/anechoic image, representing nearly full suppression of fundamental signal (gain: 85%). These settings were repeated during each injection. All studies were digitally registered as a movie clip at a rate of 7 frames per second, during 90 seconds.

**Fig 1:**
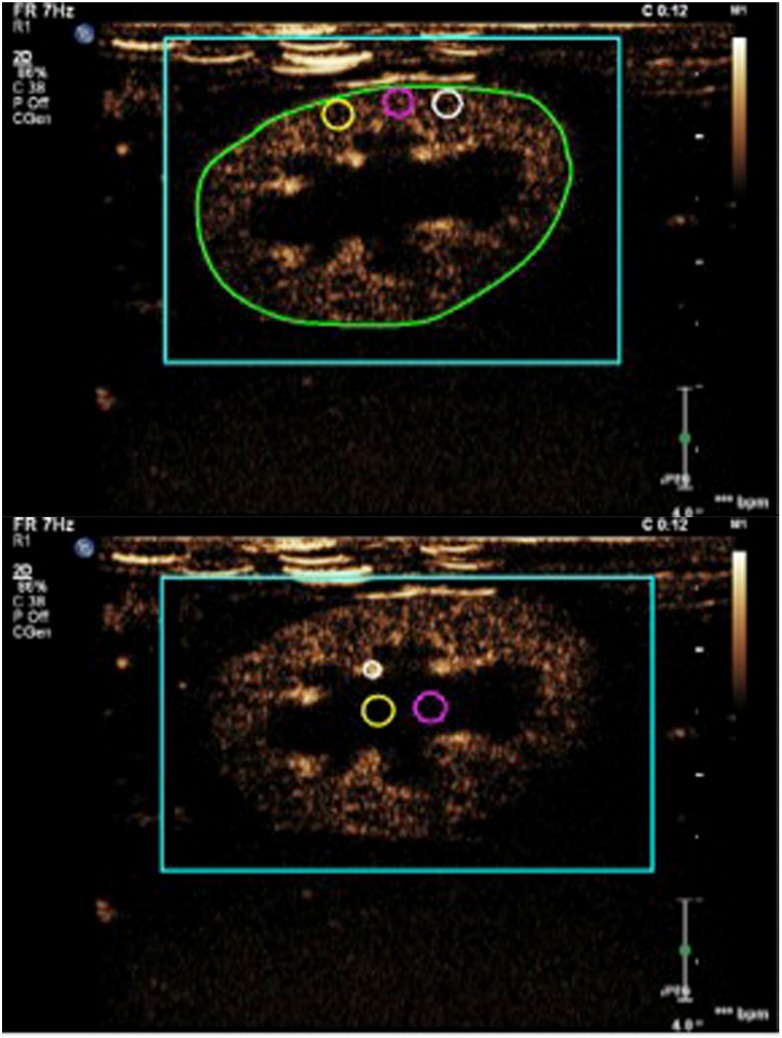
Contrast-enhanced image of the left kidney of a cat. Note the ROIs drawn around the entire kidney, in the renal cortex (top image), renal medulla and centered on an interlobar artery (bottom image).

The clips were analyzed using specialized computer software (VueBox^®^, Bracco Suisse SA, Switzerland) for objective quantitative analysis. Six regions-of-interest (ROIs) were manually drawn: 1 on an interlobar artery, 3 in the renal cortex, 2 in the renal medulla, and a ROI containing the entire kidney (Fig 1). The ROIs for the cortex and medulla were identical in size for every clip and drawn at approximately the same depth. For every ROI, the software determined mean pixel intensities proportional to contrast-agent concentration and created a time-intensity curve. Time-intensity curves were analyzed for peak enhancement (PE), wash-in area under the curve (WiAUC), rise time (RT), mean transit time (mTT), time to peak (TTP), wash-in rate (WiR), wash-in perfusion index (WiPI; WiAUC/RT), wash-out area under the curve (WoAUC), total area under the curve (AUC), fall time (FT), and wash-out rate (WoR). Parameters related to blood volume are PE, WiAUC, WoAUC and AUC. The PE corresponds to the maximum contrast medium signal intensity. The WiAUC is calculated as the sum of all amplitudes inside the range from the beginning of the curve up to the TTP. Similarly, WoAUC corresponds to the sum of all amplitudes inside the range from the TTP to the end of the descending curve. The other parameters, i.e. RT, mTT, TTP, WiR, WiPI, FT, WoR, are related to blood velocity. The WiR and WoR represent the slopes of respectively the ascending and descending curves. The RT corresponds to the time interval between the first arrival of contrast and the time of peak intensity. The FT, in contrast, is the duration of contrast wash-out. Mean transit time is the mean duration of complete contrast medium perfusion. The values for the 3 ROIs in the renal cortex and 2 ROIs in the renal medulla were averaged. Peak enhancement, and WiAUC for the cortex, medulla and entire kidney were normalized to the values obtained for the interlobar artery.

### Scintigraphy

The cats received 124 to 137 MBq (136.18 ± 7.44 MBq) of ^99m^Tc -MAG_3_ intravenously via the cephalic vein catheter, followed by a 1 mL bolus of sterile saline flush, using the 3-way stopcock.

The images were acquired using a gamma camera fitted with a low energy, high-resolution collimator. The cats were positioned in dorsal recumbency with the camera centered dorsal to the kidneys for dynamic imaging (matrix 128x128). Dynamic scanning started simultaneously with intravenous injection of the radiopharmaceutical. The dynamic protocol consisted of 60 frames at 1 second per frame, followed by 120 frames at 4 seconds per frame.

A 30 second static image of the full and empty syringe was performed. The net amount of injected radioactivity was determined by subtracting the amount of radioactivity in the syringe before and after injection measured in a dose calibrator.

**Fig 2:**
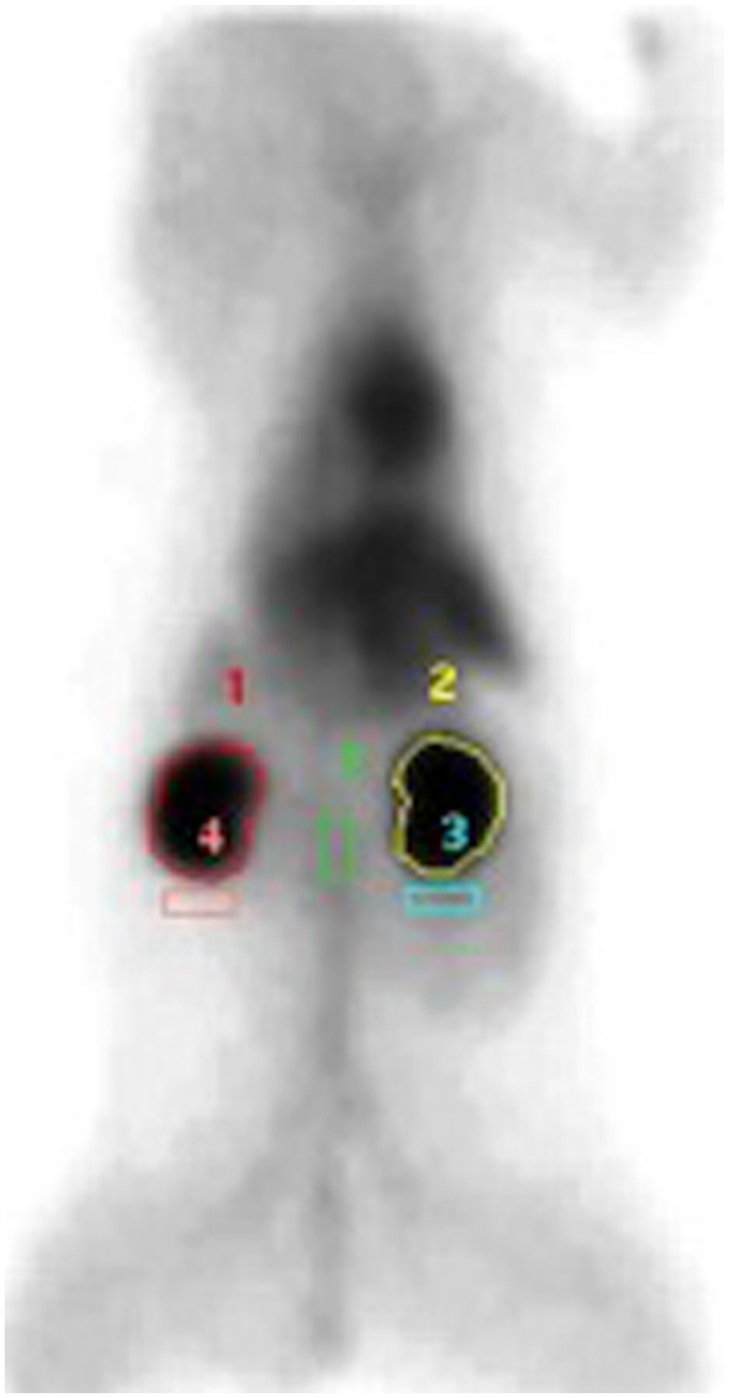
Summed dynamic dorsal images from a cat injected with ^99m^Tc-MAG_3_. Note the ROIs drawn on both kidneys with the rectangular background placed caudal to the kidneys and a rectangular ROI drawn on the aorta at the level of the kidneys.

Image frames from the dynamic acquisition for the first 30 seconds were summed. Regions of interest were manually drawn around the left and the right kidney, and the aorta at the level of the kidneys. Equally sized background ROIs were drawn caudal to the kidneys, excluding major vascular structures and the lower urinary tract (Fig 2). The regions of interest were applied to each frame and time-activity curves for the ROIs were generated. The kidney activity was background corrected. No depth correction was applied. A mathematical analysis program (Microsoft Excell) was used to determine the slopes of the kidney and aortic uptake curves.

The K/A ratio was the ratio of the initial rise of each kidney curve to the initial rise of the arterial curve [6], using the following formula:

$$\text{K}/\text{A ratio }=\frac{\text{slope of the kidney curve }}{\text{slope of the aortic curve}}$$

The flow index was calculated by using the following formula [6]:

$$\text{Flow index }=\frac{\text{Area under the aortic curve }}{\text{Area under the kidney curve}}$$

The area under the aortic curve was calculated from initial upslope to the point of peak activity. The area under the kidney curve was calculated for the same time interval.

### Statistical analysis

A mixed model with period and treatment as categorical fixed effects and cat as random effect was used (SAS Version 9.3). Analysis for the CEUS parameters was performed per location. The F-test at the 5% significance level was used to assess the effect of AT II infusion on the values of the various parameters of renal blood flow obtained with CEUS and MAG_3_.

## Results

### General

Angiotensin II infusion, administration of SonoVue^®^ and ^99m^Tc -MAG_3_ were well tolerated and no adverse effects were noticed. Good quality images were obtained with both imaging techniques in all study subjects.

The mean systolic blood pressure during the procedure was 88 ± 22 mmHg (ranging from 63 to 132 mmHg) for the AT II treatment and 72 ± 8 mmHg (ranging from 60–82 mmHg) for the placebo treatment. There was a statistically significant increase (P = 0.03) in systolic blood pressure with infusion of AT II whereas no influence of AT II infusion was noted on the heart rate.

### Contrast-enhanced ultrasound

A mean decrease of 26.1% (P = 0.04) in PE for the entire kidney and a 19.7% (P = 0.12) decrease for the renal cortex was noticed with infusion of AT II compared to control treatment. However, only the results for the entire kidney reached statistical significance.

Although not significant, a tendency for a lower AUC was observed after AT II infusion for the entire kidney and renal cortex, with the effect being most prominent for WiAUC. A 23.2% (P = 0.08) reduction in WiAUC was noted for the entire kidney, while a 21,3% (P = 0.15) reduction was present for the renal cortex. Similarly, a reduction of 18.8% (P = 0.20) for the entire kidney and 21.9% (P = 0.18) for the renal cortex for the WoAUC and a reduction of 20.7% (P = 0.14; entire kidney) and 21.8% (P = 0.14; renal cortex) in total AUC were present. None of the results reached statistical significance.

Furthermore, AT II infusion induced a significant reduction in WiPI (P = 0.04) and WoR (P = 0.02) for the entire kidney.

The results of PE and WiAUC normalized to the interlobar artery (PE* and WiAUC*) were in accordance with PE and WiAUC, i.e. a decrease of these parameters was observed with AT II infusion. However, none of these results reached statistical significance (P-values ranging between 0.34 and 0.22).

No changes were observed in any of the perfusion parameters for the renal medulla.

**Fig 3:**
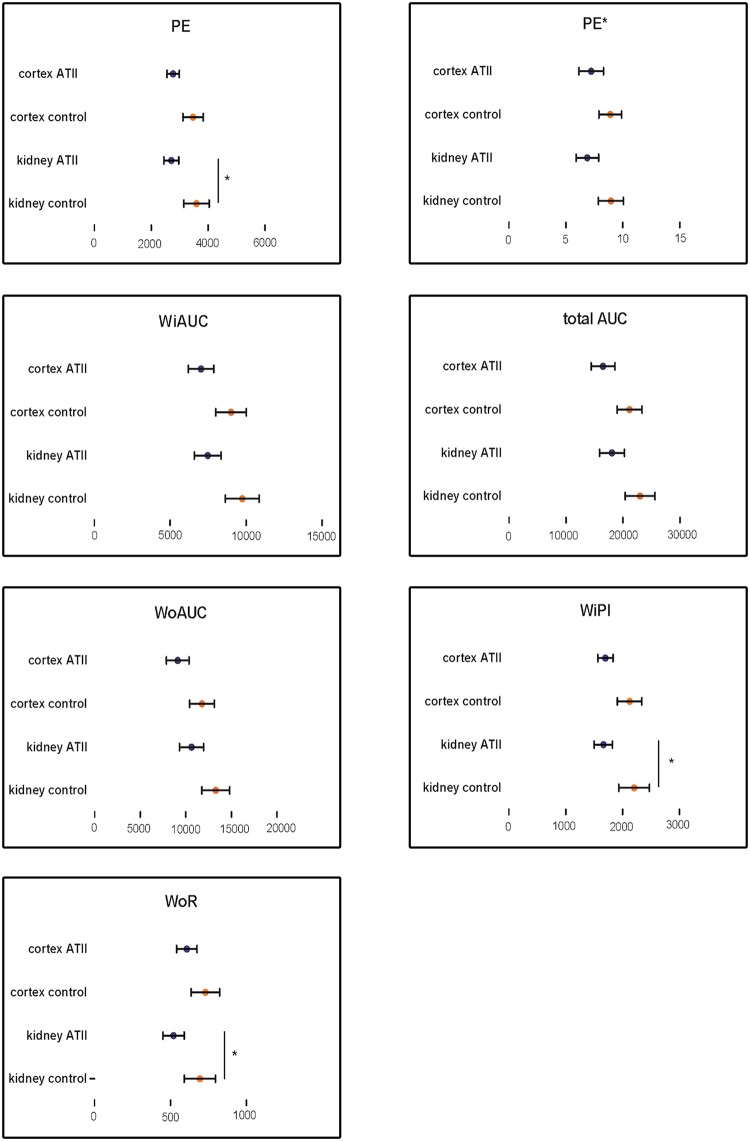
Changes in CEUS parameters induced by angiotensin II. presented as means +/- standard error. * p value < 0.05. From left to right, top to bottom: peak enhancement (PE), ratio of peak enhancement with interlobar artery (PE*), wash-in area-under-the-curve (WiAUC), total area-under-the-curve (total AUC), wash-out area-under-the-curve (WoAUC), wash-in perfusion index (WiPI) and wash-out-rate (WoR). All parameters are expressed in arbitrary units.

Graphs demonstrating the influence of AT II on the most important perfusion parameters can be consulted in Fig 3. A table containing all perfusion parameters for the entire kidney, renal cortex and medulla can be found under ‘supporting information’ (S1 Table).

### Renal scintigraphy

**Fig 4:**
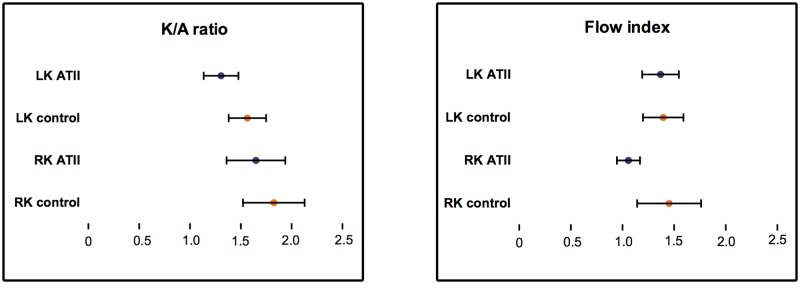
Changes in scintigraphic perfusion parameters induced by angiotensin II. presented as means +/- standard error. LK: left kidney, RK: right kidney.

A negligible effect of AT II was noticed on the flow index for the left kidney (4% decrease, P = 0.52), while a 26% (P = 0.19) decrease was noted for the right kidney. K/A ratio decreased by 16% (P = 0.22) for the left kidney and 11% (P = 0.50) for the right kidney (Fig 4, S2 Table). However, none of the parameters reached statistical significance when comparing AT II infusion with control treatment.

No association was observed between the perfusion variables obtained with CEUS and those obtained with renal scintigraphy.

## Discussion

In this study, we have shown that CEUS a promising technique is to assess perfusion changes induced by AT II infusion in healthy cats. We found a significant decrease in PE and a tendency towards a lower WiAUC compared to control measurements. Moreover a reduction in WiPI and WoR were noticed with ATII infusion.

The physiologic effects of AT II can easily explain the AT II-induced decrease in renal blood volume, noticed as a decrease in PE and AUC. Angiotensin II is a potent vasoconstrictor, causing an increased systemic blood pressure and even more important, an increased renal perfusion pressure. The latter is achieved by vasoconstriction of both the afferent and efferent glomerular arterioles [18, 19]. Consequently, AT II causes dose-dependent decreases in total and cortical renal blood flow [19, 20]. The efferent arteriole is more sensitive to the vasoconstrictive effects of AT II compared to the afferent arteriole [21]. Additionally, a decreased WiPI and WoR were noted after AT II infusion. WiPI is calculated as a ratio of WiR and RT. In this study, the reduction in WiPI is mainly caused by decrease in WiR. This corresponds to a slower inflow of contrast medium, caused by AT II-induced vasoconstriction. Similarly, the reduction in WoR corresponds to slower outflow of contrast medium.

In our study, infusion of AT II did not alter any of the perfusion parameters for the renal medulla. Studies performed on rats using invasive laser Doppler perfusion monitoring, report an inconsistent effect of angiotensin on the medullary blood flow varying from no influence to an increase in medullary blood flow [20, 22, 23]. The exact etiology remains unclear: a higher concentration of vasodilatory substances as prostaglandins, kinins and nitrogen oxide in the renal medulla, may likely cause this phenomenon [20, 23].

Our results are in accordance with a study in humans where a dose-dependent decrease in perfusion index of the renal cortex was observed with CEUS after infusion of AT II. The changes in perfusion index paralleled those in estimated renal plasma flow measured with PAH-clearance techniques [24]. In a recent study in sheep, a very heterogeneous and inconsistent response on CEUS parameters was noticed after infusion of AT II. The only significant finding was an increase in mean transit time, corresponding with a delayed replenishment, caused by a high dose AT II infusion [25]. In both the human and sheep study, CEUS was performed using continuous infusion of contrast agent followed by several destruction-refilling sequences, whereas in our study a bolus injection was performed. Therefore, different perfusion parameters are obtained in the current study compared to the human and sheep study, and thus exact comparison of the results is not possible.

The discrepancy between the flow index for the left and right kidney is most likely related to inclusion of liver activity in the ROI for the right kidney. Variable hepatic uptake of ^99m^Tc-MAG_3_ has been described in cats [26]. The anatomic location of the right kidney close to the caudate lobe of the liver complicates complicated complete exclusion of liver activity within the renal ROI.

We did not observe an association between any of the CEUS parameters and values obtained with ^99m^Tc-MAG_3_ scintigraphy. Perfusion changes induced by ATII did not reach statistical significance with renal scintigraphy. In a study with human patients with various renal diseases, a significant correlation was present between ERPF determined by PAH-clearance, ERPF determined by ^99m^Tc-MAG_3_ scintigraphy and decline ratio obtained with CEUS. However, no association was seen between the peak intensity obtained with CEUS, scintigraphy and PAH-clearance. Contrast-enhanced ultrasound in the latter study was performed using a combination of harmonic power Doppler and intermittent imaging during a continuous infusion of contrast agent [27]. A strict comparison between CEUS and renal scintigraphy is impossible because with CEUS regional blood flow is determined on a microvascular level while total renal blood flow on a macrovascular level is assessed using renal scintigraphy. Moreover, both techniques only deliver relative perfusion parameters, as no formulas exist to calculate effective or estimated renal plasma flow derived from these techniques in cats. Different perfusion parameters are calculated from both techniques, further complicating a comparison. Furthermore, there is a high heterogeneity in the parameters that are determined by CEUS. The assessed parameters depend on the injection procedure: different parameters are calculated using continuous infusing of contrast-agent compared by bolus injection. It may be assumed that constant infusion studies would suffer from less variability because constant infusions are performed using an infusion pump while bolus injections are performed manually. However, no data are available to support this hypothesis. Bolus injection has the major advantage that it is easy to administer and less cumbersome.

This study has some limitations. Although there is a gold standard for global renal plasma flow evaluation (PAH), there is not one for microperfusion. Laser Doppler probes have been used in rats for evaluation of global renal perfusion as well as cortical and medullary perfusion [20, 22, 23]. However, the technique has not been described in cats and is due to its invasiveness unethical to use. Renal scintigraphy using ^99m^Tc-MAG_3_ is the only non-invasive technique available for evaluation of perfusion in cats. Data on the use of renal scintigraphy in cats is limited and no formula is established to determine ERPF as available in dogs and human.

Second, anesthesia might suppress the effect of angiotensin II. Therefore, the response of the anesthetized cats in this study might differ from the response that would be seen in conscious cats. We choose to anesthetize the cats to eliminate the variable and unpredictable variations in blood pressure caused by stress. In a murine study, barbiturate anesthesia was found to decrease baseline arterial pressure, however it did not alter the response to AT II [28]. In sheep, isoflurane anesthesia reduced the hypertensive response to AT II both in magnitude and duration, however the reduction in renal blood flow was similar to conscious animals [29].

In this study we established a dose of AT II inducing detectable perfusion changes with CEUS, whereas no significant perfusion changes could be observed with renal scintigraphy. Future research, using multiple doses of AT II could be performed to assess the sensitivity of both techniques.

Finally, it is recommended to reduce variations in intensity depended CEUS parameters, as PE and AUC, by normalization of areas of interest with neighboring normal tissue, resulting in a ratio between the ROI and reference tissue [30, 31]. Reference tissue for the left kidney could be the abdominal aorta or the spleen. However, it is impossible to obtain a reproducible image of the kidney while simultaneously imaging the aorta or spleen in the same imaging plane. In this study, normalization to an interlobar vessel was tested. The major problem is that the interlobar vessels are also influenced by AT II infusion. Moreover, due to their small size, they are difficult to image and to correctly place a ROI, leading to relatively high variability.

## Conclusion

This study demonstrates that CEUS is a potentially valuable technique to detect changes in feline renal perfusion after infusion of AT II. These perfusion changes were not depicted by renal scintigraphy. Further research is warranted to determine the value of CEUS for diagnosis of naturally occurring diffuse renal pathology.

## Supporting Information

S1 Table Mean and Standard Errors values of renal CEUS perfusion variables of the left kidney, for the entire kidney, cortex and medulla. ^†^value represents a significant (P<0.05) effect (PE peak enhancement, PE* peak enhancement relative to interlobar artery, WiAUC wash-in area-under-the-curve, WiAUC* wash-in area-under-the-curve relative to artery, RT rise time, mTT mean transit time, TTP time to peak, WiR wash-in rate, WiPI wash-in perfusion index, WoAUC wash-out area-under-the-curve, AUC total area-under-the-curve, FT fall time, WoR wash-out rate)
(PDF)

S2 Table Mean and Standard Errors for ^99m^Tc-MAG_3_ scintigraphy parameters. Percentage uptake and kidney-to-heart ratio (K/A) for the left and right kidney separately.
(PDF)

## References

[1] Coresh J, Selvin E, Stevens LA, Manzi J, Kusek JW, Eggers P, et al Prevalence of chronic kidney disease in the United States. Jama-J Am Med Assoc. 2007;298(17):2038–47. 10.1001/jama.298.17.2038 17986697

[2] Marino CL, Lascelles BDX, Vaden SL, Gruen ME, Marks SL. Prevalence and classification of chronic kidney disease in cats randomly selected from four age groups and in cats recruited for degenerative joint disease studies. J Feline Med Surg. 2014;16(6):465–72. 10.1177/1098612X13511446 24217707 PMC4414065

[3] Lulich JP, Osborne CA, Obrien TD, Polzin DJ. Feline Renal-Failure—Questions, Answers, Questions. Comp Cont Educ Pract. 1992;14(2):127–&.

[4] Bartges JW. Chronic Kidney Disease in Dogs and Cats. Vet Clin N Am-Small. 2012;42(4):669–+. 10.1016/j.cvsm.2012.04.008 22720808

[5] Regan MC, Young LS, Geraghty J, Fitzpatrick JM. Regional renal blood flow in normal and disease states. Urol Res. 1995;23(1):1–10. 10.1007/BF00298844 7618229

[6] Daniel GB, Mitchell SK, Mawby D, Sackman JE, Schmidt D. Renal nuclear medicine: A review. Vet Radiol Ultrasoun. 1999;40(6):572–87. 10.1111/j.1740-8261.1999.tb00883.x 10608684

[7] Taylor A, Ziffer JA, Steves A, Eshima D, Delaney VB, Welchel JD. Clinical Comparison of I-131 Orthoiodohippurate and the Kit Formulation of Tc-99m Mercaptoacetyltriglycine. Radiology. 1989;170(3):721–5. 10.1148/radiology.170.3.2521734 2521734

[8] Itkin RJ, Krawiec DR, Twardock AR, Gelberg HB. Quantitative Renal Scintigraphic Determination of Effective Renal Plasma-Flow in Dogs with Normal and Abnormal Renal-Function, Using Tc-99m-Mercaptoacetyltriglycine. Am J Vet Res. 1994;55(12):1660–5. 7887507

[9] Dietrich CF, Ignee A, Hocke M, Schreiber-Dietrich D, Greis C. Pitfalls and Artefacts using Contrast Enhanced Ultrasound. Z Gastroenterol. 2011;49(3):350–6. 10.1055/s-0029-1245851 21391167

[10] Seiler GS, Brown JC, Reetz JA, Taeymans O, Bucknoff M, Rossi F, et al Safety of contrast-enhanced ultrasonography in dogs and cats: 488 cases (2002–2011). J Am Vet Med Assoc. 2013;242(9):1255–9. 10.2460/javma.242.9.1255 23600783

[11] Dong Y, Wang WP, Cao J, Fan P, Lin X. Early assessment of chronic kidney dysfunction using contrast-enhanced ultrasound: a pilot study. Brit J Radiol. 2014;87(1042). 10.1259/bjr.20140350 25060882 PMC4148829

[12] Ma F, Cang YQ, Zhao BZ, Liu YY, Wang CQ, Liu B, et al Contrast-enhanced ultrasound with SonoVue could accurately assess the renal microvascular perfusion in diabetic kidney damage. Nephrol Dial Transpl. 2012;27(7):2891–8. 10.1093/ndt/gfr789 22532616

[13] Tsuruoka K, Yasuda T, Koitabashi K, Yazawa M, Shimazaki M, Sakurada T, et al Evaluation of Renal Microcirculation by Contrast-Enhanced Ultrasound With Sonazoid (TM) as a Contrast Agent. Int Heart J. 2010;51(3):176–82. 20558907 10.1536/ihj.51.176

[14] Fischer T, Muhler M, Kroncke TJ, Lembcke A, Rudolph J, Diekmann F, et al Early postoperative ultrasound of kidney transplants: Evaluation of contrast medium dynamics using time-intensity curves. Rofo-Fortschr Rontg. 2004;176(4):472–7. 10.1055/s-2004-812992 15088169

[15] Kay DH, Mazonakis M, Geddes C, Baxter G. Ultrasonic microbubble contrast agents and the transplant kidney. Clin Radiol. 2009;64(11):1081–7. 10.1016/j.crad.2009.06.010 19822241

[16] Haers H, Daminet S, Smets PMY, Duchateau L, Aresu L, Saunders JH. Use of quantitative contrast-enhanced ultrasonography to detect diffuse renal changes in Beagles with iatrogenic hypercortisolism. Am J Vet Res. 2013;74(1):70–7. 10.2460/ajvr.74.1.70 23270348

[17] Dong Y, Wang WP, Cao JY, Fan PL, Lin XY. Quantitative Evaluation of Contrast-Enhanced Ultrasonography in the Diagnosis of Chronic Ischemic Renal Disease in a Dog Model. Plos One. 2013;8(8). 10.1371/journal.pone.0070337 23936410 PMC3731349

[18] Klein BG. Cunningham's Textbook of Veterinary Physiology. 5th ed Missouri, USA: Saunders; 2012 624 p.

[19] Evans RG, Head GA, Eppel GA, Burke SL, Rajapakse NW. Angiotensin II and neurohumoral control of the renal medullary circulation. Clin Exp Pharmacol Physiol. 2010;37(2):e58–69. 10.1111/j.1440-1681.2009.05233.x 19566838

[20] Badzynska B, Grzelec-Mojzesowicz M, Dobrowolski L, Sadowski J. Differential effect of angiotensin II on blood circulation in the renal medulla and cortex of anaesthetised rats. J Physiol-London. 2002;538(1):159–66. 11773324 10.1113/jphysiol.2001.012921 PMC2290021

[21] Itkin RJ. Effects of the Renin-Angiotensin System on the Kidneys. Comp Cont Educ Pract. 1994;16(6):753–&.

[22] Nobes MS, Harris PJ, Yamada H, Mendelsohn FAO. Effects of Angiotensin on Renal Cortical and Papillary Blood Flows Measured by Laser-Doppler Flowmetry. Am J Physiol. 1991;261(6):F998–F1006. 1721499 10.1152/ajprenal.1991.261.6.F998

[23] Walker LL, Rajaratne AA, Blair-West JR, Harris PJ. The effects of angiotensin II on blood perfusion in the rat renal papilla. J Physiol. 1999;519 Pt 1:273–8. 10.1111/j.1469-7793.1999.0273o.x 10432357 PMC2269498

[24] Schneider AG, Hofmann L, Wuerzner G, Glatz N, Maillard M, Meuwly JY, et al Renal perfusion evaluation with contrast-enhanced ultrasonography. Nephrol Dial Transpl. 2012;27(2):674–81. 10.1093/ndt/gfr345 21690200

[25] Schneider AG, Calzavacca P, Schelleman A, Huynh T, Bailey M, May C, et al Contrast-enhanced ultrasound evaluation of renal microcirculation in sheep. Intensive Care Med Exp. 2014;2(1):33 10.1186/s40635-014-0033-y 26266930 PMC4513025

[26] Drost WT, McLoughlin MA, Mattoon JS, Lerche P, Samii VF, DiBartola SP, et al Determination of extrarenal plasma clearance and hepatic uptake of technetium-99m-mercaptoacetyltriglycine in cats. Am J Vet Res. 2003;64(9):1076–80. 10.2460/ajvr.2003.64.1076 13677382

[27] Hosotani Y, Takahashi N, Kiyomoto H, Ohmori K, Hitomi H, Fujioka H, et al A new method for evaluation of split renal cortical blood flow with contrast echography. Hypertens Res. 2002;25(1):77–83. 10.1291/hypres.25.77 11924730

[28] Chapman BJ, Brooks DP, Munday KA. Half-life of angiotensin II in the conscious and barbiturate-anaesthetized rat. Br J Anaesth. 1980;52(4):389–93. 7378239 10.1093/bja/52.4.389

[29] Lee WB, Ismay MJ, Lumbers ER. Mechanisms by Which Angiotensin-Ii Affects the Heart-Rate of the Conscious Sheep. Circ Res. 1980;47(2):286–92. 10.1161/01.RES.47.2.286 7397959

[30] Tang MX, Mulvana H, Gauthier T, Lim AKP, Cosgrove DO, Eckersley RJ, et al Quantitative contrast-enhanced ultrasound imaging: a review of sources of variability. Interface Focus. 2011;1(4):520–39. 10.1098/rsfs.2011.0026 22866229 PMC3262271

[31] Tranquart F, Mercier L, Frinking P, Gaud E, Arditi M. Perfusion Quantification in Contrast-Enhanced Ultrasound (CEUS)—Ready for Research Projects and Routine Clinical Use. Ultraschall Med. 2012;33:S31–S8. 10.1055/s-0032-1312894 22723027

