# Supplementary material for: Evaluation of Feline Renal Perfusion with Contrast-Enhanced Ultrasonography and Scintigraphy
Source: PLoS One. 2016 Oct 13;11(10):e0164488. doi: 10.1371/journal.pone.0164488 (PMC5063434; doi:10.1371/journal.pone.0164488)
Supplement: S1 Table — †value represents a significant (P<0.05) effect (PE peak enhancement, PE* peak enhancement relative to interlobar artery, WiAUC wash-in area-under-the-curve, WiAUC* wash-in area-under-the-curve relative to artery, RT rise time, mTT mean transit time, TTP time to peak, WiR wash-in rate, WiPI wash-in perfusion index, WoAUC wash-out area-under-the-curve, AUC total area-under-the-curve, FT fall time, WoR wash-out rate) (PDF) [file pone.0164488.s001.pdf]

	ENTIRE KIDNEY		CORTEX		MEDULLA	
	**Control**	**Ang II**	**Control**	**Ang II**	**Control**	**AngII**
**PE**	3603.37 ± 378.55†	2663.83 ± 384.76†	3433.82 ± 299.64	2760.43 ± 306.89	999.11 ± 175.28	896.37 ±178.05
**PE***	9.04 ± 1.04	7.13 ± 1.07	8.85 ± 1.07	7.48 ± 1.09	2.80 ± 0.64	2.48 ± 0.66
**WiAUC**	9679.27 ± 1030.39	7430.28 ± 1049.89	8849.46 ± 878.01	6966.94 ± 901.73	6356.87 ± 1263.66	6534.57 ± 1289.49
**WiAUC***	11.91 ± 1.32	9.83 ± 1.29	11.08 ± 1.31	9.07 ± 1.28	9.10 ± 1.86	8.21 ± 1.82
**RT**	4.51 ± 0.37	4.64 ± 0.38	4.42 ± 0.33	4.33 ± 0.34	11.89 ± 1.56	11.70 ± 1.60
**mTT**	12.62 ± 8.42	22.89 ±8.64	9.34 ± 1.52	8.69 ± 1.56	60.33 ± 18.75	65.02 ± 19.25
**TTP**	11.92 ± 0.50	11.42 ± 0.52	12.28 ± 0.63	12.20 ± 0.64	25.75 ± 1.38	25.75 ± 1.42
**WiR**	1041.86 ± 146.02	805.77 ± 148.38	1110.44 ± 113.15	894.05 ± 114.70	136.40 ± 29.86	120.95 ± 30.50
**WiPI**	2207.41 ±231.32†	1637.87 ± 235.17†	2100.60 ± 183.63	1692.19 ± 188.14	614.88 ± 107.24	554.06 ± 108.95
**WoAUC**	13151 ± 1458.17	10673 ± 1490.23	11570 ± 1265.58	9040.66 ± 1299.76	9346.96 ± 1881.01	10349 ± 1924.66
**AUC**	22819 ± 2475.89	18101 ± 2527.11	20838 ± 2062.22	16301 ± 2117.92	15695 ± 3106.91	16887 ± 3175.71
**FT**	6.35 ± 0.82	7.00 ± 0.84	5.74 ± 0.37	5.69 ± 0.39	20.25 ± 4.68	20.93 ± 4.77
**WoR**	703.78 ± 89.52†	501.20 ± 90.35†	730.60 ± 83.27	606.07 ± 84.07	85.66 ± 22.13	77.24 ± 22.61

Table 1. Mean and Standard Errors values of renal CEUS perfusion variables of the left kidney, for the entire kidney, cortex and medulla †value represents a significant ($P<0.05$) effect
