# Supplementary material for: Evaluation of Feline Renal Perfusion with Contrast-Enhanced Ultrasonography and Scintigraphy
Source: PLoS One. 2016 Oct 13;11(10):e0164488. doi: 10.1371/journal.pone.0164488 (PMC5063434; doi:10.1371/journal.pone.0164488)
Supplement: S2 Table — Percentage uptake and kidney-to-heart ratio (K/A) for the left and right kidney separately. (PDF) [file pone.0164488.s002.pdf]

	LEFT KIDNEY		RIGHT KIDNEY	
	**Control**	**Ang II**	**Control**	**Ang II**
**% uptake**	5.16 ± 0.51	5.53 ± 0.50	5.60 ± 0.42	5.90 ± 0.41
**K/A**	0.15 ± 0.02	0.16 ± 0.02	0.15 ± 0.02	0.16 ± 0.02

Table 2. Mean and Standard Errors for ^{99m}Tc-MAG$_3$ scintigraphy parameters: percentage uptake and kidney-to-heart ratio (K/A) for the left and right kidney separately.
